# Supplementary material for: Characterization of the Dynamic Gastrointestinal Digests of the Preserved Eggs and Their Effect and Mechanism on HepG2 Cells
Source: Foods. 2023 Feb 13;12(4):800. doi: 10.3390/foods12040800 (PMC9955911; doi:10.3390/foods12040800)
Supplement: Supplementary file 1 [file foods-12-00800-s001.zip › foods-2092320-supplementary.pdf]

Supplementary materials

S.1 The dynamic human gastrointestinal -IV (DHGI-IV)

The DHGI-IV (Figure S1) consists of two main components, a bionic stomach and small intestine. The bionic stomach is a 3D print of a real human stomach, with the geometry, size, internal folds and other physiological structures of a real stomach. The peristaltic contraction process of the stomach wall is simulated by physical movements such as roller squeezing, similar to the contraction process of the real stomach wall. In the small intestine component, a rolling squeeze device simulates the peristaltic and segmental movement processes in different parts of the small intestine. Moreover, the rate of secretion of simulated gastric and intestinal fluids is precisely controlled by the syringe pump, and together they complete the digestive process in the gastrointestinal tract.

S.2 In vitro gastric emptying assay of PED

During digestion, the PED from the stomach was collected and weighted every 15 min for the calculation of the gastric emptying rate. In order to characterize the difference in the gastric emptying between the solids and liquids, the weights of both the supernatant (liquid fraction) and pellet (solid fraction) in the emptied gastric digesta were determined after centrifugation (14,000 g for 20 min at 4°C). The ratio of the amount of solids and liquids in the collection to the total amount of solids and liquids is then calculated separately to give the retention ratio in the stomach. For a further quantitative comparison of the gastric emptying characteristics of the PED, their retention ratio data were fitted with the modified Elashoff's power-exponential model. The model has been widely used to describe solid and liquid gastric emptying, and is described as:

$$y(t)=1-(1-e^{-kt})^{\beta} \quad (1)$$

where y(t) is the fractional meal retention at time t in minutes, k is the gastric emptying rate per minute, and β is the extrapolated y-intercept from the terminal portion of the curve. The parameters of k and β for each trial were estimated using the non-linear least squares method.

The half-time ($t_{1/2}$) of gastric emptying was calculated from Eqn (1). when $y(t) = 0.5$, and it could be deduced as:

$$t_{1/2} = -1/k \times ln\left(1 - 0.5^{1/\beta}\right) \quad (2)$$

Then, the lag phase time (t_{lag}), which is defined as the time to achieve the maximum rate of gastric emptying, after ingestion of the PED was calculated by assuming that the 2nd derivative of the equation (1) equals to zero:

$$t_{lag}= ln\beta/k \quad (3)$$

S.3 Determination of antioxidant activity

S.3.1 Determination of DPPH scavenging rate

The sample solution (2.0 mL) was mixed with 2.0 mL of DPPH in anhydrous ethanol (0.1 mmol/L). The mixture was stood for 30 min at room temperature in the dark. Subsequently, the absorbance was determined at 517 nm with UV spectrophotometer (Nanodrop2000/2000c; Thermo Scientific, Pittsburgh, PA, USA). Anhydrous ethanol was used in the blank instead of DPPH solution, while anhydrous ethanol was used for the control, instead of sample solution. The ability to scavenge DPPH free radicals was calculated using the following equation:

$$\text{DPPH Radical scavenging capacity (\%)} = (A_1 - A_2)/A_0 \times 100 \quad (4)$$

Where A_1, A_2 and A_0 represent the absorbance of the sample, blank and control, respectively.

S.3.2 Determination of ABTS scavenging rate

The $ABTS^+$ was previously prepared by mixing 7 mM ABTS with 2.45 mM potassium persulfate ($K_2S_2O_8$) and kept in the dark at room temperature for 16 h. Then the $ABTS^+$ solution was diluted in deionized water to an absorbance of 0.70 ± 0.02 at 734 nm. The sample solution was mixed with the ABTS reagent at the volume ratio 1:10, incubated at 25°C in the dark for 6 min. The absorbance values were measured at 734 nm using a UV spectrophotometer. Distilled water was used as a blank. The ability to scavenge ABTS radical was calculated using following formula:

$$\text{ABTS Radical scavenging capacity (\%)} = (A_0 - A_i)/A_0 \times 100 \quad (5)$$

Where A_0 and A_i represent the absorbance of the control and sample, respectively.

S.3.3 Determination of hydroxyl radical scavenging rate

The hydroxyl radical was generated through a Fenton reaction (2.0 mL of 6 mM $FeSO_4$ and 2.0 mL of 24 mM H_2O_2). After 4.0 mL of sample solution added, mixture volume was made up to 25 mL with 8 mM salicylic acid in absolute ethanol. The mixture was incubated at 37°C for 30 min in a water bath, and the absorbance was read at 510 nm with a UV spectrophotometer. Distilled water was used instead of H_2O_2 as a control. The scavenging activity was calculated using the following formula:

$$\text{Hydroxyl radical scavenging capacity (\%)} = (A_0 - A_1)/A_0 \times 100 \quad (6)$$

Where A_0 and A_1 represent the absorbance of the control and sample, respectively.

S.3.4 FRAP determination

The reagent for ferric ion reducing antioxidant power (FRAP) assay was freshly prepared by mixing 10 mM TPTZ, 12 mM ferric chloride, and 0.3 M sodium acetate (pH 3.6) at 1:1:10 volume ratios. The assay was performed by mixing sample solution and FRAP reagent at the volume ratio of 1:30 and incubated in the dark for 6 min. After the reaction, absorbance value was measured at 593 nm by a UV spectrophotometer.

Table S1 Preparation of stock solutions of simulated digestion fluids.

Constituent (mmol/L)	SSF	SGF	SIF
KCl	15.10	6.90	6.80
KH_2PO_4	3.70	0.90	0.80
$NaHCO_3$	13.60	25.00	85.00
NaCl	——	47.20	38.40
$MgCl_2(H_2O)_6$	0.15	0.10	0.33
$(NH_4)_2CO_3$	0.06	0.50	——
$CaCl_2(H_2O)_2$*	1.50	0.15	0.60

* To prevent calcium precipitation, aqueous $CaCl_2$ solution was added during the preparation of the mock digest.
SSF: simulated salivary fluid; SGF: simulated gastric fluid; SIF: simulated intestinal fluid.

Table S2 The operating parameters of the DHGI-IV system.

Indicators to control	Operating parameters
Peristalsis frequency of stomach	3 times/min
Injection speed of SGF	2.9 mL/min
Opening size of pylorus	0-2 mm
Opening frequency of pylorus	1 time/2 min
Injection speed of SIF	1 mL/min
Peristalsis frequency of intestine	6 times/min
Peristalsis frequency of duodenal	12 times/min
Stomach tilt angle	0-30 min: 0°；30-90 min: -15°；90-120 min: -30°
Digestion time	Stomach digestion: 2 h; Intestinal digestion: 4 h
Temperature	37℃

Table S3 Sequence of primers in RT-qPCR.

Primer Names	Primer Sequence	Product Length/bp
Homosapeins β-actin	F: 5'-CACGATGGAGGGGCCGGACTCATC-3'<br>R: 5'-TAAAGACCTCTATGCCAACACAGT-3'	240 bp
Homosapeins Bcl-2	F: 5'-GCCTTCTTTGAGTTCGGTGG-3'<br>R: 5'-GAAATCAAACAGAGGCCGCA-3'	192 bp
Homosapeins Bak	F: 5'-GGGACGACATCAACCGAC-3'<br>R: 5'-CAGAAGAGCCACCACACG-3'	152 bp
Homosapeins VEGF	F: 5'-GGAGGAGGGCAGAATCATCA-3<br>R: 5'-CTTGGTGAGGTTTGATCCGC-3'	247 bp
Homosapeins HIF-1α	F: 5'-TCCAAGAAGCCCTAACGTGT-3'<br>R: 5'-TGATCGTCTGGCTGCTGTAA-3'	180 bp

Table S4 Parameters of *k, β,* $t_{1/2}$ and t_{lag} are obtained by fitting the *in vitro* gastric retention data with the modified Elashoff's model.

Meal form	*k*	*β*	$t_{1/2}$	t_{lag}	*r*
Solid fraction	0.0463±0.0012	19.46±1.14	72.3±0.43	64.1±0.51	0.992
Liquid fraction	0.0278±0.0010	3.21±0.99	46.1±6.7	36.4±7.2	0.989
PED	0.029±0.0014	4.02±0.05	63.5±2.1	48±1.2	0.994

PED: preserved egg digests.

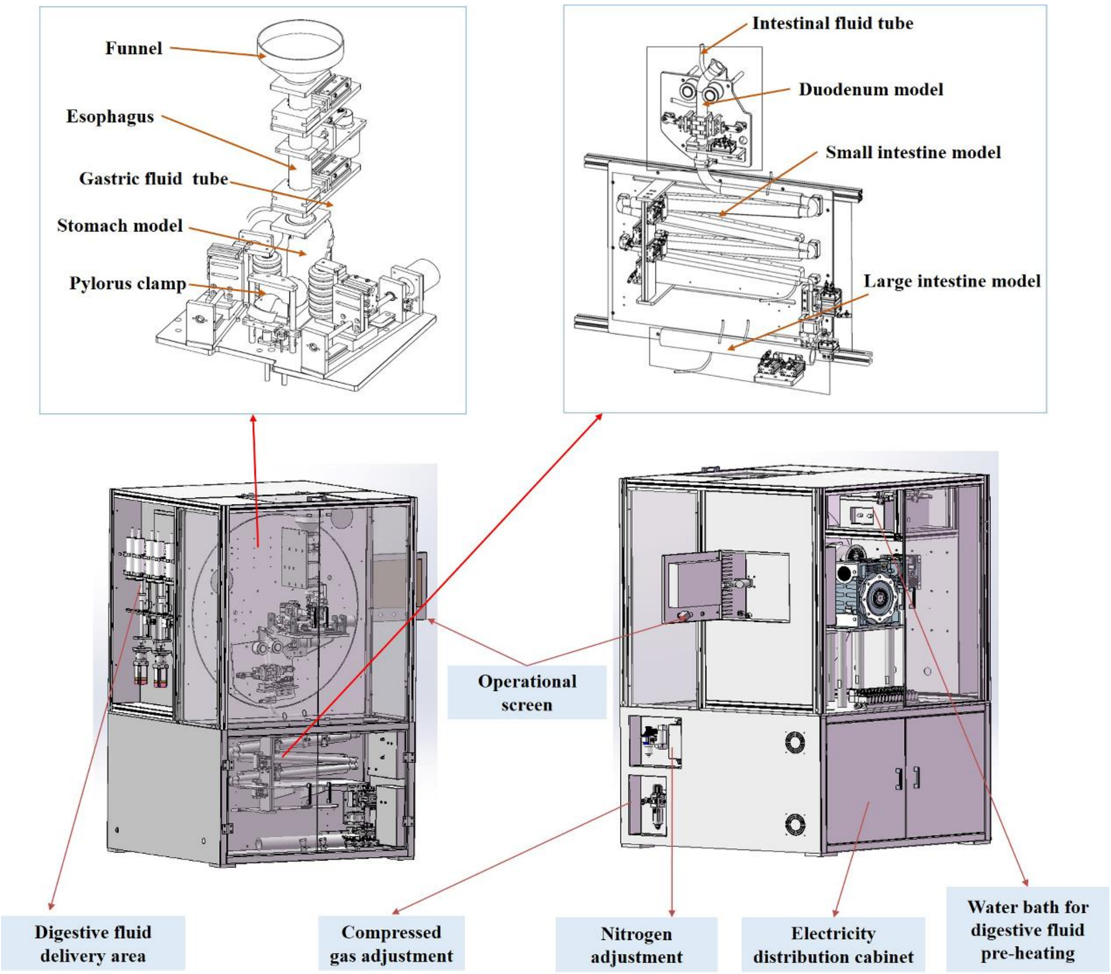

Figure S1 The in vitro dynamic human gastrointestinal -IV (DHGI-IV)
